# Prevalence and phenotype of eating disorders in assisted reproduction: a systematic review

**DOI:** 10.1186/s12978-022-01341-w

**Published:** 2022-02-07

**Authors:** Marine Le Floch, Anaïs Crohin, Philippe Duverger, Aline Picard, Guillaume Legendre, Elise Riquin

**Affiliations:** 1 Department of Child and Adolescent Psychiatry, Centre Hospitalier Universitaire d’Angers, Angers, France; 2 Department of Perinatal Psychiatry, Centre Hospitalier Universitaire de Lille, Lille, France; 3 Department of Obstetrics and Gynecology and Medically Assisted Reproduction, Centre Hospitalier Universitaire d’Angers, Angers, France; 4 University of Angers, University of Nantes, LPPL, SFR Confluences, 49000 Angers, France; 5 University Service of the Fondation Santé des Étudiants de France, Sablé sur Sarthe Clinic, Paris, France; 6 Pediatric Psychiatry Department, University Hospital of Angers, 4, Rue Larrey, 49933 Angers Cedex 9, France

**Keywords:** Assisted reproductive technique, Eating disorders, Infertility, Psychiatry

## Abstract

**Background:**

Eating disorders (EDs) are common conditions that mainly affect women of reproductive age and have a major impact on fertility. Our systematic review focuses on the prevalence of EDs in patients in the process of assisted reproductive technique (ART) and describes the phenotypes of EDs identified.

**Methods:**

Our systematic review is based on the PRISMA criteria. Articles were collected using the Medline/Pubmed, Web Of Science and Cochrane databases. The articles chosen had to mention the prevalence of ED in infertile patients undergoing ART and be cohort or case–control studies assessing the prevalence of ED during fertility treatment.

**Main findings:**

Fifteen articles were included in this review. The prevalence of active ED varied between 0.13 and 44% depending on the types considered in each study. The main phenotypes described were EDNOS (eating disorder not otherwise specified) and binge eating disorders (BED) occurring in women with a normal body mass index (BMI) and a history of ED. Mainly subthreshold forms with cognitive distortions were described.

**Conclusion:**

This review highlights a 6 times higher prevalence of EDs in infertile patients undergoing fertility treatment compared to regular pregnant women. However, diagnosing these conditions is complex. As a result, it is essential that professionals in contact with this population are alert to symptoms consistent with these conditions in order to refer them to specialized psychiatric care.

## Plain English Summary

Eating disorders are frequent pathologies that primarily affect women of childbearing age. Numerous articles reveal an increased risk for the mother and the child in case of an active disorder during pregnancy.

We conducted a systematic review to determine the prevalence and phenotypes of eating disorders in infertile subjects undergoing fertility treatment.

The results of the fifteen articles included show a prevalence six times higher than in pregnant women in the general population. Subjects with eating disorders have normal body mass indexes. The active forms are mainly characterized by episodes of binge eating disorders or other unspecified forms. Studies also describe incomplete forms characterized by the presence of dysfunctional thoughts around shape and weight without associated compensatory behavior.

Professionals working in the field of reproductive medicine and providing fertility treatment have a major role to play in identifying and referring these subjects at risk to specialized care.

## Résumé

**Introduction:**

les troubles des conduites alimentaires (TCA) sont des pathologies fréquentes affectant principalement les femmes en âge de procréer avec un impact majeur sur la fertilité. Notre revue de la littérature s’intéresse à la prévalence du trouble chez les sujets inscrits dans un processus d’assistance médicale à la procréation (AMP) et décrit les phénotypes des TCA repérés.

**Méthode:**

notre revue de la littérature se base sur les critères PRISMA. Les articles ont été collectés en utilisant les bases de données Medline/Pubmed, Web Of Science et Cochrane. Les articles sélectionnés devaient faire état de la prévalence des TCA chez les sujets infertiles recourant à une AMP, être des études de cohorte ou cas-témoins évaluant la prévalence des TCA au cours d’une AMP.

**Résultats:**

quinze articles ont été inclus dans cette revue. Les prévalences de TCA actifs variaient entre 0,13 et 44% en fonction des formes considérées. Les principaux phénotypes décrits étaient les formes non spécifiques (EDNOS) et hyperphagiques (BED) survenant chez des femmes avec un indice de masse corporel (IMC) normal et présentant des antécédents de TCA. Il était décrit des formes subsyndromiques prédominées par des distorsions cognitives.

**Conclusion:**

cette revue met en évidence une prévalence de TCA supérieure chez les sujets infertiles recourant à une AMP par rapport aux femmes enceintes en population générales. Leur diagnostic est cependant complexe. Il est donc capital que les professionnels au contact de cette population soient vigilants aux symptômes évocateurs afin d’orienter vers une prise en charge spécialisée.

## Background

Eating disorders (EDs) are severe conditions which mainly affect women [1]. Although they have been discussed for a long time, it is only since the 80s that they have been considered as a psychiatric pathology. Since then, the definition of these disorders has continued to evolve. The DSM-5 categorizes EDs into seven types, defining them as follows: “*feeding and eating disorders are characterized by a persistent disturbance of eating or eating-related behavior that results in the altered consumption or absorption of food and that significantly impairs physical health or psychosocial functioning*” [2]. EDs usually appear during adolescence, or at beginning of adult life. Currently, the prevalence of lifetime EDs in women in the general population varies between 8 and 10%, with a peak incidence in women at the beginning of reproductive age, at the end of the adolescence [1].

Recent studies carried out on women suffering from EDs report difficulties to control their fertility [3, 4]. Infertility has often been described in women with current EDs [5, 6]. It may occur at different stages of the weight-loss process, and it may persist after weight recovery. The mechanisms are partially known. They mainly involve the hypothalamic-pituary-gonadal axis. Recent works on hormonal and neuroendocrine pathways involving leptin, ghrelin or the corticotropic axis, helps to explain the impact of variations in energy intake on the central nervous system [7–10].

Several studies, including Easter et al., 2011, found that women suffering from EDs were twice as likely than the general population to have received fertility treatment or assisted reproductive techniques (ART) [11–13]. Similarly, Bye et al., and a French cohort of midwives highlight the difficulties of identifying EDs [14, 15]. Due to the variability in clinical presentations and the difficulties in diagnosing EDs, there is a high risk of underestimating the real prevalence of this condition.

Indeed, international guidelines recommend early and multidisciplinary treatment of EDs in order to improve the maternal and fetal prognosis [5, 16]. Early identification during the fertility treatment could be of major interest for both mother and fetus. Therefore, we conducted a systematic review of the literature. Our primary outcome was to investigate the prevalence of EDs in women seeking fertility treatment. The secondary outcome of this review is to describe the phenotypes of these EDs.

## Materials and methods

We performed a systematic review according to the PRISMA guidelines [17].

Between March and July 2021, a search of three databases (MEDLINE, Cochrane and Web of Science) was perform using a combination of key words such as infertility, assisted reproductive technique, feeding and eating disorders and the different type of EDs. The datasets supporting the conclusions of this article are available in the the PubMed repository, [https://pubmed.ncbi.nlm.nih.gov], the Cochrane library [https://www.cochranelibrary.com], and Web Of Science [https://www.webofscience.com]. As an example, for the PubMed search, the search term was defined as follows: (("Reproductive Techniques, Assisted"[MH]) OR (“Assisted reproductive technology” [TIAB]) OR (“ART” [TIAB]) OR ("Infertility"[MH]) OR (“infertility”[TIAB])) AND (("Feeding and Eating Disorders"[MH]) OR (“eating disord*”[TIAB]) OR ("Anorexia"[MH]) OR ("Anorex*"[TIAB]) OR ("Bulimia"[MH]) OR ("Bulimia"[TIAB]) OR ("Binge-Eating Disorder"[MH]) OR ("Binge-Eating Disorder"[TIAB])) along with a “human” search filter. No limits were applied with regards to publication date. This algorithm was adapted for each database. Gray literature was also consulted.

Selected articles followed inclusion criterias:

- Assess in their primary or secondary objective the prevalence of EDs, or present results allowing the calculation of a prevalence of EDs during fertility treatment
- be medical articles from quantitative cohort or case control studies
- be published in French or English

Articles were excluded if they: did not meet the inclusion criteria; investigated the prevalence of ART in patients with EDs; studied infertile females, without providing information on whether or not they received infertility treatment; were reviews, meta-analyses, case reports, or case study; did not allow a prevalence to be calculated.

The bibliographies of the excluded articles have been analysed to ensure that no relevant references were ignored.

Articles were reviewed for eligibility through titles, then abstracts and subsequently full texts if relevant. The articles included underwent standardized critical analysis of their methodology, using the STROBE checklist. However, no references were excluded from his analysis.

Double-blind research was conducted by AC. The results were compared and discussed in order not to ignore any reference.

The results were summarized in a table. Main characteristics were then analyzed in a descriptive synthesis.

## Results

**Fig. 1:**
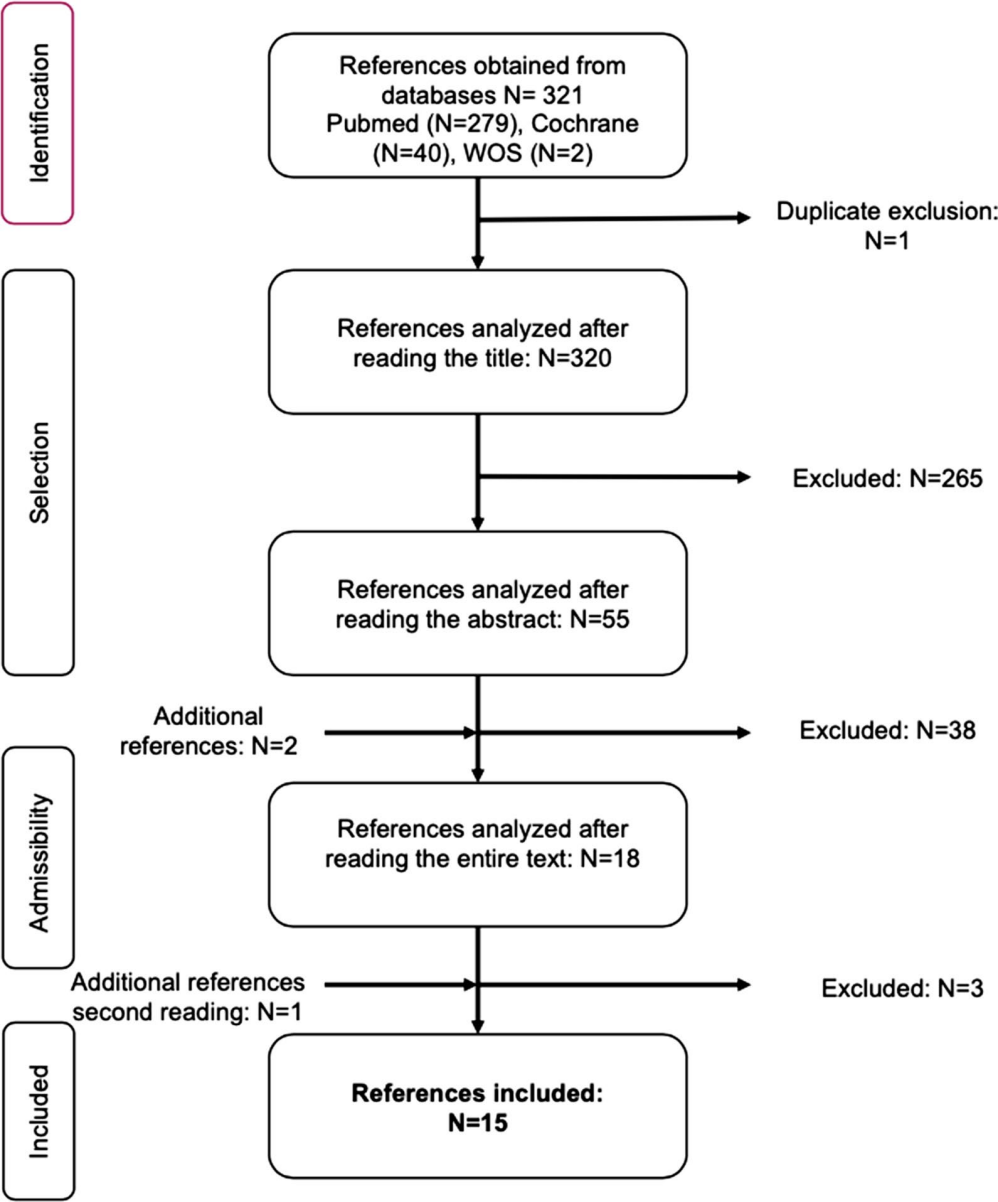
Flow chart

Database searches revealed three hundred and twenty-one articles. The flow chart describes the selection process (Fig. 1). The second reading revealed 5 points of disagreement. One of these articles was included in our analysis. The other four references were excluded because they investigated the prevalence of ART in patients with EDs which was one of our exclusion criteria. Thus, we obtain a strong agreement between the investigators with a Kappa index of 0.76.

Overall, fifteen references were included. Two articles were selected by the analysis of the bibliographies. Their characteristics and results of interest are given in Table 1. The studies included were mainly anglo-saxon (N = 7) or nordic (N = 3).

**Table 1:** summary of the articles included in the systematic review

Author, date	Sample population	Assessment	Key findings
Abraham et al. 1990 [18]	*Obj*: study of diet. hist. and of physical exercise in IF receiving GnRH stimulation *Inclusion criterion*: secondary amenorrhea – Failed OI using clomiphene citrate – N = 14	– DSMIII-R – ED assessed: AN, BN – semi-structured interview – anthropometric measurements: weight, size, skin fold (4 sites) – Quest.: EAT, EDI	*Prev.*: 92.8% hist. of ED (N = 13) of which: *77% AN (N* = *10), 7.7% AN* + *BN (N* = *1), 7.7% BN (N* = *1), 7.7% atypical (N* = *1)* – 35.7% “active atypical form” (N = 5) *Phen.*: 84.6% normal BMI (N = 11) – 57% follow-up of ED (N = 8) – Weight control method: restriction + diet 57% (N = 8), physical exercise 43% (N = 6), vomiting 21% (N = 3), laxatives 7% (N = 1) *Comorb*.: depression (N = 2)
Assens et al. 2015 [31]	*Obj.*: assessing prev. of ED in IF undergoing ART *Inclusion criterion*: – DANAC cohort – N = 42,915 *Exclusion criterion*: – IUI	– ICD-8, ICD-10 – ED assessed: AN, BN, EDNOS – diagnostic coding of the Danish Psychiatric Central Research Register (hospitalisations + consultations)	*Prev*.: 0.63% LTP of severe episode of ED (N = 271) of which: *41% AN (N* = *111) with 32% experiencing atypical forms of AN, 35% BN (N* = *95) with 20% experiencing atypical BN, 24% EDNOS (N* = *65)* *Phen*.: – 83.4% ED known before resorting to MAP – Ovulatory infertility* – More frequent use of IVF or ICSI*
Barbosa et al. 2020 [29]	*Obj.*: assessing prev. of ED in IF treated receiving GnRH stimulation *Inclusion criterion*: – < 43 years old – IHH, N = 21 – Control group with other type of infertility N = 21 *Exclusion criterion*: – Non-Francophone – male infertility – active psychiatric disorder	– ICD-10, DSM IV-TR – ED assessed: AN, BN, BED – Quest.: QSD, CIDI	*Prev.*: – IF with IHH: 95.2% LTP ED (N = 20) of which: *85% AN (N* = *19), 5% BN (N* = *1)* – Control group: 23.8% LTP ED *restrictive type AN (N* = *5)* *Phen*.: – BMI IF IHH lower compared to other types of infertility* – Normal BMI in control group
Bruneau et al. 2017 [8]	*Obj.*: assessing prev. of ED in an IF population receiving MAP *Inclusion criterion*: – French speakers – N = 60 *Exclusion criterion*: – Male infertility	– DSM-IV – ED assessed: AN, BN, BED – Screening: SDQ, SCOFF, FKW, BSQ, HAD, FertiQol – Diagnosis: MINI Module H	*Prev.*: 17% LTP ED (N = 10) of which: *50% hist. AN (N* = *5), 20% hist. BN (N* = *2), 10% AN (N* = *1), 10% BN (N* = *1), 10% hist. BED (N* = *1)* *Phen.*:—average BMI and ideal BMI similar to non-ED* – ambivalence sc. for desire for a child: positive correlation with body image concerns, negative correlation with BMI – 60% SCOFF positive with ED* *Comorb.*:—higher level of anxiety *vs* non-ED* – altered quality of life (emotional and physical aspects)
Christin-Maitre et al. 2006 [28]	*Obj.*: assessing effectiveness of Tx using pulsatile GnRH release *Inclusion criterion*:—IHH N = 248	– Declarative	*Prev.*:—45.8% hist. of ED (N = 113): *40.5% hist. AN (N* = *100), 5.3% hist. BN (N* = *13)* *Phen.*:—normal average BMI – 52.2% (N = 109) of IF make food selection – 9% (N = 16) physical activity > 5 h/week
Cousins et al. 2015 [19]	*Obj.*: comparison of ED sympt. in IF *vs* females in the general pop *Design*: cross-sectional comparative study *Inclusion criteria*:—[18–44 years old], English speakers – No comorbidity – Unexplained infertility N = 51 – Control group in general care N = 34	– DSM-IV-TR – ED assessed: AN, BN, EDNOS (including BED) – Quest.: EDI-3-RF and SC, Herman and Polivy revised restraint scale, STAI, BDI-II	*Prév.*:—27.5% hist. of ED (N = 14) – 13.7% active episodes (N = 7) *Phen.*:—normal average BMI* – Pursuit of slimness sc. and BN higher in IF vs non-ED* – Diet. restriction sc. lower in IF *vs* non-ED* – Body dissatisfaction sc. lower in IF *vs* non-ED*
Freizinger et al. 2010 [30]	*Obj.*: assessing prev. of ED in IF *Design*: comparative study *Inclusion criterion*:—before hormonal induction using FSH – N = 82	– DSM-IV – ED assessed: AN, BN, EDNOS (including BED) – Tel. interview: SCID module H-Eating disorder, SDQ – Quest.: EDE-Q, IPAQ, lifestyle	*Prev.*: 20.7% LTP ED (N = 17) of which: *41% hist. AN (N* = *7), 29% active EDNOS (N* = *5) of which 80% BED (N* = *4), 12% hist. BN (N* = *2), 18% hist. EDNOS (N* = *3)* *Phen.*: normal BMI > 50%, compared to non-ED*, no low BMI – Total EDE-Q sc. higher *vs* non-ED* – Diet. concerns sc. for size and weight higher *vs* non-ED + general population* – Diet. restriction sc. of ED comparable to non-ED + general population* – 76.4% of ED not disclosed to doctor
Langley 2014 [20]	O*bj.*: designing a nutritional screening questionnaire for IF *Inclusion criterion*:—before 1st medical visit – N = 300	– No baseline – ED assessed: NS – Declarative	*Prev.*:—4.1*% hist.* of ED (N = 10) – 1.4% active ED (N = 4) *Phen.*:—21.8% dietary restrictions without ED (N = 55) – 81% want to lose weight before their first medical check – 40% “unrealistic” weight loss goal of which 7.1% with BMI < 18.5 – Methods used: diets (49%), physical activity “active” (29%), or even “very active” (13%)
Resch et al. 1999 [23]	*Obj.*: assessing prev. of ED in IF and diet. habits in IF *Inclusion criterion*:—functional infertility – N = 75	– DSM-IV – ED assessed: AN, BN, BED – Quest.: SDQ, BCDS, ANIS, EDI, BITE, BDI	*Prev.*: 44% active ED (N = 33) of which: *49% subclinical forms of BN (N* = *16), 27% BN (N* = *9), 15% infraclinical forms of AN (N* = *5), 9% AN (N* = *3)* Phen.:—episodes of binge eating: > 1 per month (N = 8), > 1 per week (N = 8) – Increased prevalence of anovulation in women with subclinical BN *Comorb.*: 64% depression in IF (N = 49)
Rodino et al. 2016 [24]	*Obj.*: assessing psychological wellbeing in relation to the BMI of obese IF *Inclusion criterion*: – [20–47 years old] – N = 403 *Exclusion criteria*:—gamete donation, preimplantation diagnosis, maintenance of fertility	– DSM-IV – ED assessed: AN, BN, BED – Quest.: SDQ, IPAQ, DASS-21, PANAS, FPI, RSES, CPQ, EDE-Q	*Prev.*:—5.7% hist. of ED (N = 23) – 0.7% active episode (N = 3) *Phen.*:—74% normal BMI with ED – Obese females (*indep. of PCOS status*): BED higher*, lower level of self-esteem*, increased concerns about body shape and perfectionism
Rodino et al. 2016 [21]	*Obj.*: assessing prev. of ED in IF *Inclusion criteria*:—[20–40 years old], English speakers – ovulation follow-up, OI, IUI, IVF, ICSI – N = 385 *Exclusion criteria*:—gamete donation, preimplantation diagnosis, oncology – No medical consultation	– DSM-IV – ED assessed: AN, BN, BED – Quest.: SDQ, EDE-Q, IPAQ	*Prev.*:—6.8% hist. of ED (N = 26) - 1.6% active ED (N = 6) *Phen.*:—BMI at top end of normal - average EDE-Q sc. higher with hist. ED* - ovulation disorder *vs* other type of infertility: higher overall score and subscales EDE-Q*, prone to compulsive eating with loss of control, method of weight control by vomiting, laxatives, compulsive physical activity at a sustained rate* - 23% ED not disclosed to doctor
Sbaragli et al. 2008 [22]	*Obj.*: psychiatric assessment of infertile couples before medical intervention *Design*: prospective case–control study *Inclusion criterion*:—1st ART cycle – N = 81 couples – control group: T3 pregnancy, N = 70 couples	– DSM-IV – ED assessed: BED – Assessment of partners: SDQ, SCID-I	*Prev.*:—18% active BED (N = 15) – 11% hist. of ED (N = 9) *Phen*.: BED and hist. BED more common in PCOS and functional infertility*
**Stewart** et al., 1990 [25]	*Obj.*: assessing prev. of ED in IF *Inclusion criterion*:—N = 66	– DSM-III-R – ED assessed: AN, BN, EDNOS – Screening: SDQ, EAT-26 – Diagnosis: non standardised	*Prev.*: 16.7% active ED (N = 11) of which: *55% EDNOS (N* = *6), 36% BN (N* = *4), 9% AN (N* = *1)* *Phen.*: SD to determine ideal comparable weight comparable regardless of ED status
**Volgsten** et al., 2008 [26]	*Obj.*: assessing prev. of psychiatric disorders in IF and IM *Inclusion criteria*:—IVF or ICSI – N females = 413 – N males = 412 *Exclusion criterion*:—gamete donation	– DSM-IV – PRIME-MD	*Prev*: 0.2% active EDNOS type ED (N = 1) in IF *Phen.*:—normal average BMI in females with ED *Comorb.*:—depression 10.9% (N = 45) – Comorb. in IF: 2 diag. 36.2% (N = 46), ≥ 3 diag. 7.9% (N = 10)
**Yli-Kuha** et al., 2010 [27]	*Obj.*: assessing psychiatric morbidity in IF receiving ART *Design*: comparative cohort study *Inclusion criteria*:—IVF, ICSI, FET – infertile group N = 9175 – control group general pop. N = 9175	– ICD-8, ICD-9, ICD-10 – ED assessed: NS – Diagnostic coding of psychiatric disorders from end of hospitalisation records	*Prev.*: 0.13% hospitalization for ED (N = 12) of which: *67% before intervention (N* = *8), 33% after intervention (N* = *4)* *Phen.*:—no significant difference in number of IF hospitalizations *vs* non-ED – fewer hospitalizations for ED in infertile patients if Tx is successful* – no influence on the parity of hospitalizations for ED

*ANIS* anorexia nervosa inventory scale, *BCDS* bulimic cognitive distortions scale, *BDI-II* Beck depression inventory II, *BITE* bulimia investigatory test, Edinburgh, *BSQ* body shape questionnaire, *CIDI* composite international diagnostic interview, *CPQ* clinical perfectionism questionnaire, *comorb.* comorbidity, *DASS-21* depression anxiety and stress scale 21 items, *diag*. diagnosis, *Diet.* dietary, *EDE-Q* eating disorder examination questionnaire, *EDI-3-RF* Eating Disorder Inventory-3-Referral Form, *EDI-3-SC* Eating Disorder Inventory-3-Symptom Checklist, *FET* frozen embryo transfer, *FKW* desire to have a child questionnaire (fragebogen zum kinderwunsch), *FPI* fertility problem inventory, *HAD* hospital anxiety and depression scale, *hist*. history, *IF* infertile female(s), *IVF* in vitro fertilisation, *ICSI* intracytoplasmic sperm injection, *IHH* idiopathic hypogonadotropic hypogonadism, *IM* infertile male(s), *IPAQ* international physical activity questionnaire, *IUI* intrauterine insemination, *LTP* lifetime prevalence, *MINI* mini international neuropsychiatric interview, *NS* non specific, *OI* ovulation induction, *PANAS* positive and negative affect schedule, *phen*. phenotype, *pop*. population, *prev*. prevalence, *PRIME-MD* primary care evaluation of mental disorders, *quest*. questionnaire, *RSES* Rosenberg self-esteem scale, *sc* score, *SCOFF* sick-control-one-fat-food, *SD* standard deviation, *SDQ* socio-demographic questionnaire, *STAI* Spielberger state trait inventory, *sympt*. symptoms, *tb* trouble, *tel*. telephone, *Tx* treatment

*Statistically significant results, p < 0.05

### Prevalence

The prevalence of active EDs was found to be between 0.13 and 35.7%. This rate increased to 44% if subthreshold forms were associated to complete active forms [18–27].

Articles describing a history of EDs found a prevalence ranging from 4.1 to 92.8% [18–22, 24, 28]. The three studies carried out on infertile females receiving GnRH stimulation, reported elevated histories of EDs, ranging from 45.8 to 95.2% [18, 28, 29]. Barbosa et al., found that women suffering from infertility of hypothalamic origin were found to have a history of ED that was four times higher than those in the control group with another type of infertility [18].

Four articles focused on the lifetime prevalence of EDs in infertile women [8, 29–31]. The prevalence in those cases was found to be between 0.2 and 95.2%. Only Assens et al. found that the prevalence of lifetime EDs was lower in infertile females undergoing fertility treatment than in the general population (0.63% vs 0.73%, p = 0.025) [31].

### Phenotypes

#### Body mass index

Ten studies looked at body mass index (BMI) [8, 18–21, 24, 26, 28–30]. Nine of these found normal BMI values (18.5–25 kg/m^2^) in women suffering from EDs. BMI values were not statistically different to those of women not suffering from an ED, nor from those in the general population. The study carried out by Cousin et al. noted that although women with EDs had normal BMI, these were significantly lower than for those in the control group (p = 0.019) [19].

Barbosa et al. found that the BMI of infertile females was normal, except for those whose infertility was of hypothalamic origin (23.1 vs 18.1 kg/m^2^). The differences in BMI values between the two groups was statistically significant. Women with infertility of hypothalamic-pituitary origin also reported statistically lower BMI minima than the other types of infertility (15.7 vs 19.8 kg/m^2^) [29].

Sbaragli et al. in 2008, found BMI which were in the normal to high range (23.8–25 kg/m^2^) or even in the overweight range, for women suffering from infertility caused by polycystic ovarian syndrome (PCOS) before undergoing fertility treatment [22].

#### Types of EDs

The EDs studied in the articles varied depending on the standards used (DSM III to IV-TR). Studies that reported lifetime prevalence and history of EDs identified a predominance of a history of anorexia nervosa in infertile women [8, 18, 28–31]. When considering only current EDs, the most common ED was eating disorder not otherwise specified (EDNOS), including binge eating disorders (BED) [18, 22, 25, 30].

Three studies specifically focused on women suffering from functional infertility of hypothalamo-pituitary origin with hormonal stimulation with GnRH [18, 28, 29]. These studies focused on women suffering from infertility of hypothalamo-pituitary origin, either functional or genetic [18]. They found a predominance of active anorexia nervosa in these women (95.2%, or 20 out of 21 women). Barbosa et al. was the only one to use a control group, which included women with other types of infertility. The EDs identified in the control group were also of the restrictive anorexia nervosa type [29].

Sbaragli et al., found a significant association between BED, or a history of BED, and PCOS [22]. Rodino et al. found that obese women, independently of their PCOS status, had a significantly higher risk of suffering from a BED (OR 7.9, CI (3.421–18.312), p < 0.001) [24]. In another article, Rodino et al. found that there was a tendency towards food compulsions in women with ovulatory disturbances. This study also reported that infertile females were more likely to use weight-control measures such as induced vomiting and laxatives. They also had a significantly higher probability of engaging in high-intensity physical activity (OR 6.98, CI (1.39–34.90), p = 0.018) [21]. Other studies were consistent with these results [18, 19, 28, 30].

Sbaragli et al. investigated the association between BED and infertility in men. The results found no significant difference between infertile males, and the fertile control group [22].

#### Cognitive patterns and dysfunctional thoughts

Using the EDE-Q questionnaire, the studies showed that the infertile females’ scores were significantly higher in the ED groups than in the non-ED groups for the following factors: perfectionism; a drive for thinness; and eating, weight and shape concerns [8, 19, 21, 24, 30]. These factors were found at significantly higher rates in ovulatory infertility [21].

Eighty-one percent of infertile women, regardless of their ED status, reported a desire to lose weight before their first medical consultation. 40% percent of them reported “unrealistic” weight loss goals, which, in 7.1% of cases would lead to underweight [20].

In contrast, the studies carried out by Cousins et al. and Freizingher et al. showed that dietary restraint and concerns were either similar, or significantly lower than for women without EDs or women from the general population. These women also had lower body dissatisfaction scores [19, 30].

With regards to the desire for a child, Bruneau et al. showed that a low BMI and high body shape concern score were associated with an ambivalent desire to have a child [8].

#### Comorbidities and follow-up

Volgsten et al. found that 30.8% (N = 127) of infertile females had a psychiatric diagnosis, and that this number was 10.2% (N = 42) for infertile males. It also found that several psychiatric comorbities were also common in infertile subjects. 36.2% of women had two associated comorbidities, and 7.9% had three or more [26].

With regards to anxiety, Bruneau et al. found that women with an ED had a significantly higher level of anxiety than those without an ED [8]. Sbaragli et al. found that infertile females were more likely to have a history of anxiety disorders [22]. However, Cousin et al. did not find any significant differences between the two groups with regards to the anxiety trait or state [19].

With regards to depression, statistical analyses did not find any significant differences between women with and without EDs [8, 19].

Yli-Kuha et al. identified a lower number of hospitalizations for EDs when ART resulted in a pregnancy that went to full term. The number of children did not seem to affect the number of hospitalizations [27].

Before undergoing the treatment procedures, 83% of women were aware of their EDs diagnosis [31], and 57% stated that they benefited from psychiatric follow-up [18]. Volgsten et al. found that 20.7% (N = 23) of subjects suffering from a psychiatric pathology benefited from psychiatric treatment (psychotherapy and/or treatment with medications) [26]. Langley et al. found that only one-third of women accepted a dietary counselling offered to them after risk factors for ED were identified [20].

Freizinger et al. reported that 76.4% of women did not disclose their disorder to their physician [30]. Rodino et al. also found low levels of disclosure [21].

#### Type of infertility and art procedure

Infertile females with an ED had significantly more ovulatory or functional infertility. Resch et al. identified that incomplete forms of bulimia were also associated to anovulation [23]. Works carried out on infertility of hypothalamo-pituitary origin have reported a regular resumption of cycles after returning to a “normal” weight, or hormonal stimulation [18, 28, 29]. According to Assen et al., in-vitro fertilizations or intracytoplasmic sperm injections were significantly more frequent with ovulatory disorders. Women with EDs underwent significantly fewer treatment cycles than women without EDs (between 1 and 3 cycles, p = 0.035) [31].

## Discussion

To our knowledge, this systematic review is the first one to discuss the prevalence and phenotypes of EDs in infertile females undergoing fertility treatment. There are few studies that deal specifically with the prevalence of EDs with ART (N = 9).

The studies’ findings regarding the prevalence of EDs (between 0.13 to 95.2%) were highly variable and depended on whether the focus was placed on current, past, or lifetime EDs. The difficulty in carrying out a robust comparative analysis can be explained by the differences in methodology between the studies, as well as by their respective biases. Inclusion criteria (age, gender, sexuality) and fertility treatment strategies differ according to the country. In Nordic studies, registries exclude women who underwent artificial insemination [26, 27, 31]. However, when associated with ovulation induction, this is an effective first-line treatment for functional infertility [32]. This explains the very low rate of EDs in Nordic cohorts. Similarly, the study conducted by Yli-Kuha et al. focused on hospitalizations, leading to the identification of only the most extreme clinical situations [27]. It is therefore possible to infer that the prevalence of EDs in infertile females in those countries is higher than that found in the studies.

We notice an important heterogeneity in the diagnostic criteria for EDs in the studies. This includes standards changes (ICD, DSM), evolution between the DSM-III and the DSM-IVTR and heterogeneity in tools to detect EDs. Moreover, these tools are used in the absence of validated questionnaire which can be used in the peripartum period [33]. Finally, the quality of the methodology of some of the articles is debatable. Two of the articles conform to fewer than half of the STROBE checklist’s quality criteria for observational studies [18, 20]. Apart from the two national cohort studies, the study sample sizes were small with a significant attrition rate up to 33% [23, 26, 30].

Although the values of the results which appear in our review are difficult to analyze, the orders of magnitude of the prevalence are substantially higher than those found in pregnant women. The literature states that the prevalence of current EDs in pregnant women is between 5.1 and 7.5%, i.e. up to six times less than in our population of interest [34].

With regards to the phenotype of current EDs in infertile women, they are mainly EDNOS or BED forms. These are associating episodes of binge eating and compensatory activities (sustained physical exercise) for weight-control purposes. This data agrees with studies of EDs in pregnant women [34–36]. Several studies in our review found subthreshold types of EDs [19, 21, 23, 30]. Subsyndromal types of EDs include body image perturbations, with some body dysmorphia, and without any associated compensatory activities [26]. In a study carried out by Fassino et al. in 2008, which excluded infertile females with a known ED, questionnaires showed some cognitive patterns in common between infertile women and women with anorexia nervosa. These characteristics included feelings of inadequacy, insecurity and fears related to maturity. Interpersonal disturbances are significantly more frequent in those experiencing functional infertility. However, they did not include altered attitudes or behavior with regards to food [37]. The presence of those cognitive patterns is significantly correlated with a higher risk of obstetrical and neonatal complications. Notably, a lower Apgar score at five minutes has been described in newborn babies born to women with subsyndromal EDs [3].

In their work, Bruneau et al. described ambivalence to desire for a child in women with high body concerns. This result needs to be substantiated by regression analyses while also considering potential confounding factors such as maternal depression. Nevertheless, this ambivalence is an important factor to consider when establishing the parent–child relationship. As a matter of fact, the existence of a maternal ED during pregnancy increases the risk of postpartum depression and can represent an obstacle to the establishment of an early bond [38]. Mothers with an ED report greater difficulties in determining their child's needs. There is a risk that women with restrictive anorexia nervosa will project their eating and body concerns into their child [36]. Newborns of mothers with an active ED during pregnancy show difficulties in maintaining their homeostasis when faced with stress factors. These children also face more difficulties in emotional regulation as they grow up. A neurodevelopmental pathway has been put forward to explain this vulnerability. The pathway incorporates hormonal, metabolic and epigenetic mechanisms linked to maternal pathological eating behaviors during the period of synaptic formation and myelination in the third trimester of pregnancy [39].

Multiple studies in our review observed that women do not disclose their history of ED to their doctor even though their symptoms may still be active. This problem can affect up to three quarters of infertile females with an ED [30]. This problem is well documented in the literature [13, 40]. In 2018, Bye et al. assessed the obstacles for diagnosing EDs in pregnant women. Firstly, there are obstacles that are an intrinsic part of the disorder such as denial or lack of desire to change [15]. In the specific case of infertility treatment, some women report not passing on information about their ED because they were unaware of the impact it could have on fertility [30]. Moreover, the fear of stigma affects the disclosure to healthcare professionals. The stigma of mental health is widely acknowledged, but it may be even greater in the case of EDs. Indeed, sufferers are incorrectly seen as being more responsible and in control of their eating behavior than the general population [41]. In tandem, health professionals appear to have little confidence in their ability to identify EDs. Furthermore, women and healthcare professionals report that there is a lack of opportunity and time in routine antenatal care to openly discuss EDs [15].

As a result, checking for EDs symptoms in infertile females seeking fertility treatment is a complex task. Healthcare professionals who carry out preconception assessments have a major role to play in the monitoring and referral of patients at risk of an ED. Including brief screening techniques for eating disorders in the assessment could be useful [21]. The use of the 5-items rapid questionnaire SCOFF (Sick, Control, One, Fat and Food) has been recommended [42]. This tool offers good sensitivity for screening for anorexia nervosa and bulimia. In contrast, it is less sensitive for screening BED and using it in overweight populations is less reliable [43].

In 2019, Paslakis et al. updated a screening algorithm based on the 2009 version by Andersen and Ryan. They recommend using the shortened 8-item version of the Eating Attitude Test (EAT-8) because of the good positive predictive value, in combination with anthropometric measurements such as weight and BMI. However, the EAT-8 only exist in a German validated version. As a result, the use of the SCOFF questionnaire remains an interesting alternative. Having carried out our review, it seems important to systematically include a check for significant weight loss or gain and intensity of physical activity while determining patient history. In addition, it seems relevant to regularly question dysfunctional thoughts related to diet, morphology or ambivalence regarding the desire for a child. These proposals are in line with international recommendations that emphasize the importance of early psychiatric care in order to reduce the risk of post-partum symptoms [5, 16]. It has been described that the symptoms of EDs remain present in pregnant women with an easing of symptoms during the first trimester and a potential upsurge at the end of pregnancy or postpartum [11, 33, 44]. While Yli-Kulha et al. report significantly fewer cases of hospitalization after a pregnancy resulting from fertility treatment, these results should be qualified by the reluctance to consent to hospitalization that separates the mother and child at an early stage.

The strength of our review lies in its methodological quality, which is based on PRISMA criteria. We enabled the identification of a maximum number of articles by using a broad search equation and relatively unrestricted exclusion criteria. The use of several databases also enabled the collection of additional references.

Our study presents several obstacles for the generalizability of the results. Firstly, as described above, the various articles included in the review have inherent methodological limitations with assessment and recruitment biases. The heterogeneity of the results prevents quality meta-analysis from being performed. In addition, the small number of specific studies dealing with the prevalence of EDs in fertility treatment led us to include studies where the raw results allowed us to calculate a prevalence. This choice constitutes a bias, as the prevalence figures calculated do not benefit from statistical analysis assessing the impact of confounding factors.

## Conclusion

Our systematic review highlights a very high prevalence of active EDs among infertile women seeking fertility treatment. The prevalence in this population is up to 6 times higher than in regular pregnant women.

Our study describes non-specific or incomplete forms of EDs characterized by cognitive distortions centered on body image and diet, without compensatory behaviors being systematically associated. These forms are less obvious clinically and they are more complex to detect for non-psychiatric health professional. Nevertheless, they present significant obstetrical, neonatal, and psychological morbidities for both mother and child, which justify their early detection. It is therefore a major challenge for fertility professionals to be aware of this condition in order to be able to use specific screening strategies and to offer appropriate care.

Future research remains necessary to develop screening tools for pregnant women in order to gauge more precisely the burden of EDs in this population. Moreover, an assessment of the needs could be of major interest to set up information and prevention campaigns for screening for EDs.

## Data Availability

The datasets analysed during the current study are available in the PubMed repository, [https://pubmed.ncbi.nlm.nih.gov], the Cochrane library [https://www.cochranelibrary.com], and Web Of Science [https://www.webofscience.com] or by mailing the corresponding author on reasonable request.

## Abbreviations

ED: Eating disorders
ART: Assisted reproductive technics
BMI: Body mass index
EDNOS: Eating disorder not otherwise specified
BED: Binge eating disorder
PCOS: Polycystic ovarian syndrome
